# Assessing Brain Neurophysiology in COVID-19 Patients With Prolonged Cognitive Fatigue: A Comparison With Persistent Post-concussion Symptoms

**DOI:** 10.7759/cureus.88160

**Published:** 2025-07-17

**Authors:** David S Oakley, Mo Mortazavi, Daniella K Rivera, Leila Samsam, Taylor P Seitz, Leslie Streeter

**Affiliations:** 1 Electrophysiology, WAVi Research, Boulder, USA; 2 Trauma, Sports Medicine, Rehabilitation, and Concussion Center (SPARCC), Tucson, USA; 3 Trauma, Tucson Medical Center, Tucson, USA; 4 Family Medicine, David Grant Medical Center, Andrew Taylor (A. T. Still University School of Osteopathic Medicine, Fairfield, USA; 5 Trauma, University of Arizona, Tucson, USA

**Keywords:** cognitive fatigue, concussion, electroencephalogram, event related potentials, long covid

## Abstract

Introduction: Brain fog and cognitive dysfunction are frequently reported in post-viral fatigue syndromes such as long COVID, yet these symptoms remain challenging to quantify objectively. Notably, many individuals with long COVID describe clinical features that overlap with those observed in patients with persistent post-concussion symptoms (PPCS), including cognitive fatigue, exertional intolerance, mood disturbances, visual and balance problems, headaches, and neck pain. Emerging evidence suggests that PPCS is associated with distinct electrophysiological abnormalities, including altered functional connectivity (measured by electroencephalography (EEG) coherence), spatial changes in EEG amplitude distribution (notably increased frontal alpha and left-right asymmetry), and reduced cognitive evoked potentials (e.g., the auditory P300 response). In this study, we investigated whether these electrophysiological markers of PPCS are also present in individuals experiencing post-viral fatigue following COVID-19, aiming to provide objective measures to better characterize and quantify cognitive impairment in this population.

Materials and methods: Thirty-one patients (mean age 45 ± 9 years) reporting persistent neurocognitive symptoms following COVID-19 infection (10 ± 2 months post-infection) were evaluated at intake to a brain injury clinic while seeking assessment/treatment for prolonged cognitive complaints. Over this time period, 64 PPCS patients (69% female; age: 42 ± 11 years) were evaluated at the same clinic for concussion-related symptoms using identical protocols. These were compared to seventy age-matched controls (mean age 45 ± 5 years) without a history of COVID-19 or neurological conditions. Assessments comprised standard concussion assessments with symptom profiles that included cognitive fatigue and EEG with event-related potentials (ERP). We then compared the EEG and ERP metrics known to be sensitive to declines in mental performance (i.e., PPCS) for both the long COVID group and age-matched controls.

Results: The long COVID cohort demonstrated neurophysiological alterations paralleling those observed in PPCS, including significantly impaired processing speed and reduced physical reaction times compared to controls (p < 0.001), as well as cognitive electrophysiological deficits, such as attenuated P300 amplitudes, which reflect impaired attention allocation (p < 0.001). These biomarkers normalized concomitantly with symptom resolution at long-term follow-up (mean 20 ± 8 months post-baseline assessment).

Conclusions: Our data demonstrate that ERP deficits, characteristic of cognitive decline in conditions like concussion, PPCS, and aging, are replicable in long COVID patients. Notably, these electrophysiological abnormalities (reduced P300 amplitude and altered coherence) correlate with quantifiable cognitive fatigue and functional neurological impairments. Biomarker normalization tracked with symptom resolution at follow-up (20 ± 8 months), confirming their clinical relevance. These accessible metrics provide objective parameters to identify virally induced cognitive deficits, addressing a critical need for validated diagnostic tools in post-viral syndromes.

## Introduction

A substantial proportion of individuals who have experienced COVID-19 develop persistent complications, collectively referred to as long COVID or post-acute sequelae of SARS-CoV-2 infection. These complications commonly include general fatigue and cognitive dysfunction, often described as brain fog [1-2]. Quantifying brain fog and fatigue remains challenging; current assessments rely primarily on symptom inventories and self-report questionnaires regarding memory impairment, reduced mental clarity, poor concentration, subjective feelings of disorientation, headaches, and confusion.

Numerous neuroimaging studies have identified structural and functional abnormalities in the brains of individuals who have had COVID-19 [3-5]. Proposed mechanisms underlying these persistent symptoms include ongoing neuroinflammation, vascular complications, and hypoxia, among others. A longitudinal study of patients who underwent neuroimaging both before and after infection demonstrated alterations in diffusion measures, which serve as proxies for tissue damage, as well as reductions in gray matter thickness. In another relevant study of long COVID patients with persistent cognitive symptoms, including memory problems, brain fog, increased fatigue, and reduced quality of life, cognitive test performance did not significantly differ from that of controls at two years post-infection. However, MRI analysis revealed notable differences, including decreased brain volumes and reduced cortical thickness in several brain regions, among the COVID-19 group [6].

Despite the insights provided by neuroimaging studies, the absence of standardized neuroimaging protocols in routine clinical practice for long COVID means that diagnosis and management often depend heavily on patient self-report. This reliance can leave both patients and clinicians uncertain about the underlying causes of symptoms and how best to address them. The resulting ambiguity is reflected in the literature, where estimates of the prevalence of persistent brain fog among COVID-19 patients vary widely, ranging from as low as 7% to as high as 70% [7].

This work aims to compare the clinical features of long COVID with those of persistent post-concussion symptoms (PPCS), both of which manifest with overlapping symptom profiles. While most mild traumatic brain injuries (mTBI) resolve within approximately one month, an estimated 10-30% of affected individuals experience persistent symptoms beyond this period, resulting in PPCS. Similar to long COVID, PPCS is characterized by brain fog, fatigue, memory impairment, reduced mental clarity, poor concentration, subjective feelings of disorientation, headaches, and confusion [8]. As is the case with long COVID, the absence of standardized neuroimaging protocols in routine clinical practice and the reliance on self-reported symptoms have led to PPCS being frequently attributed to psychosocial factors. Notably, up to 40% of patients with PPCS are referred for psychological evaluation [9].

Neuroimaging studies demonstrate that structural, functional, and/or metabolic alterations occurring acutely after mTBI can persist in PPCS [9]. While neuroimaging elucidates physiological mechanisms in PPCS and long COVID, its clinical implementation remains limited by practicality constraints. In contrast, electroencephalography (EEG) with event-related potentials (ERPs), which are EEG responses time-locked to sensory/cognitive stimuli, offers greater clinical accessibility for the longitudinal monitoring of cognitive impairment [10-13]. Patients with mTBI exhibit electrophysiological deficits, such as reduced ERP amplitudes, in both the acute and chronic phases of post-concussive syndrome (PCSS). Notably, PPCS presents unique electrophysiological pathologies distinct from acute-phase abnormalities [12-13].

In this study, we retrospectively analyze patient data collected during routine clinical evaluations, representing real-world, heterogeneous populations that self-report symptoms of PPCS or long COVID. Our primary research question is whether EEG/ERP abnormalities observed in PPCS, including metrics associated with reduced cognitive performance (CP), are also present in long COVID. Given the overlapping symptom profiles and confounding psychosocial factors in both conditions, we designed this comparative study to identify more objective neurophysiological markers that complement clinical findings. We hypothesize that, to the extent these symptoms have a physiological basis, brain function analysis via EEG/ERP will reveal similarities between PPCS and long COVID. Specifically, if subjective complaints such as “brain fog” reflect underlying physiological dysfunction, we expect to observe reduced ERP amplitudes in both patient groups, even if the specific pathophysiological mechanisms differ.

## Materials and methods

Study design

This is a retrospective analysis of data collected during clinical evaluations. These evaluations included EEG with ERP, which is the primary focus of this study. Here, we rely on clinician assessments to determine the diagnostic categories and historical data, collected with the same methods, for the control group. Solutions Institutional Review Board issued approval 1MAY14-48

Study population

Thirty-one patients (50% female; age: 45 ± 9 years) with no history of concussion but presenting with persistent long COVID symptoms were evaluated at a specialized PPCS clinic. Assessments occurred at 10 ± 2 months post-infection, with longitudinal follow-up at 20 ± 8 months post-initial evaluation. Over this period, 64 PPCS patients (69% female; age: 42 ± 11 years) with no history of viral fatigue were evaluated at the same clinic for concussion-related symptoms using identical protocols. Tests included the CP symptom screening tool (scale of 0-3, none-to-severe) [14], a battery of visual testing that included vestibular-ocular reflex, and the WAVi Scan (trail making test (TMT), reaction time, and EEG collected during an audio oddball ERP protocol) [12-13]. Several studies have examined the patterns of relations between trail-making and health, where TMT completion times have been seen to decline with concussion and cognitive impairment [12,15]. Physical reaction time also represents a straightforward measurement that correlates with a host of conditions, including concussion [16].

The non-long COVID, non-PPCS control group consisted of 70 WAVi assessments from healthy individuals (mean age: 45 ± 5 years; 55% female), derived from three previous studies [17-19]. Participants were measured during wellness exams across three independent clinics using identical EEG/ERP and TMT protocols, software, and hardware as those used for the PPCS and long COVID evaluations described above.

Subjects who had a yield of less than 80% on evoked responses due to artifacts were excluded from the study for all groups. In addition, those who had a history of head trauma, fatigue (including viral fatigue), or psychiatric conditions, all related to PPCS and long COVID, were excluded from the control group.

EEG acquisition and extraction

**Figure 1:**
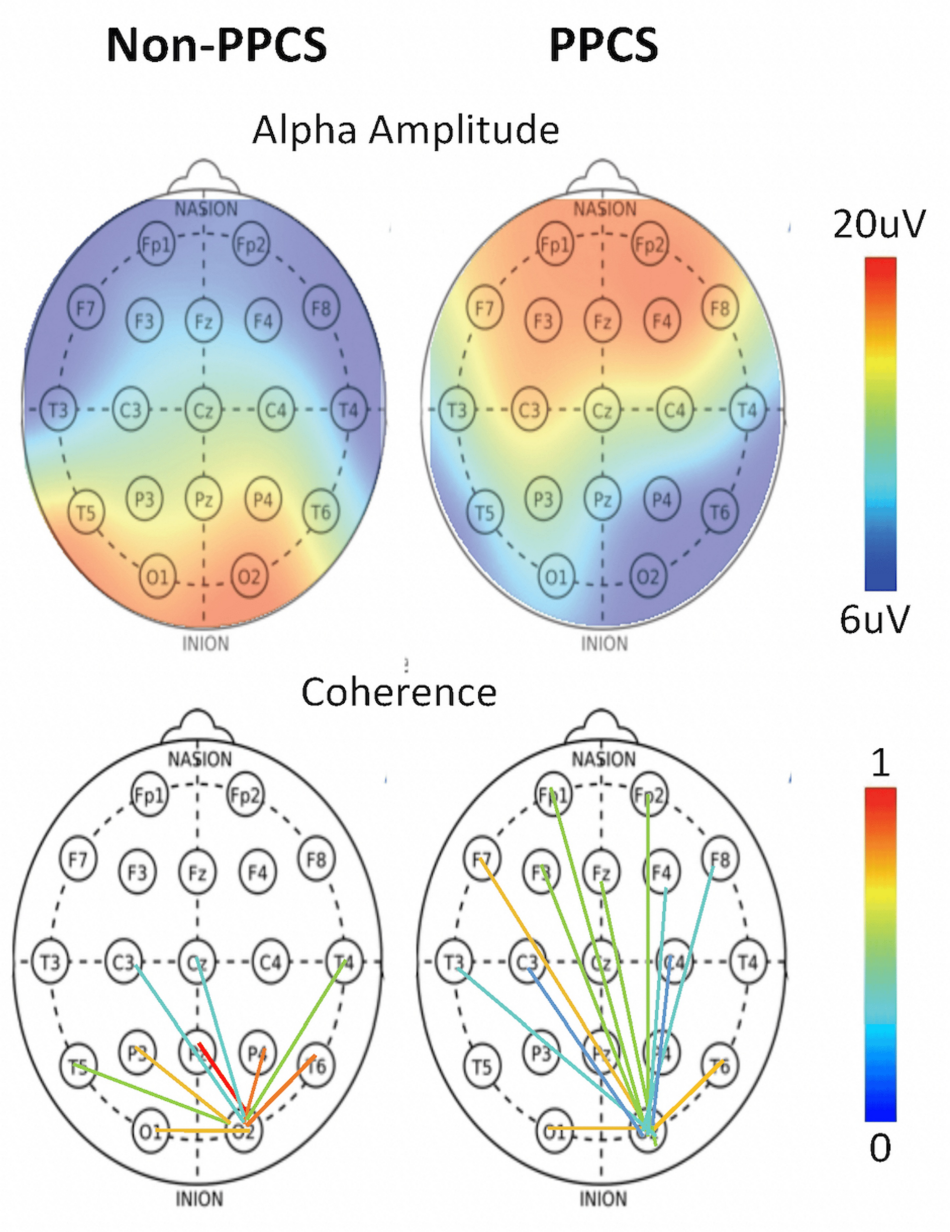
(Top) Alpha amplitude as a function of scalp location in typical non-PPCS and PPCS patients. (Bottom) Occipital connectivity from the O2 electrode as a function of scalp location in typical non-PPCS and PPCS patients PPCS: persistent post-concussion symptoms Image Source: Mortazavi et al., 2023 [13]. Published with permission. Open access.

The EEG was recorded using FDA-cleared WAVi Scan 1.1 (WAVi CO, Denver, CO, USA), sampled at 250 Hz and bandpass filtered between 0.5 and 30 Hz. The electrodes were placed according to the International 10-20 system (Figure 1), and linked reference electrodes were placed at the earlobes. Test administrators were instructed to establish electrode impedances below 30 kW for EEG locations and below 20 kW for the ground-to-ear locations where possible. These targets are well below the 1 GW input impedance of the WAVi amplifiers, are practical in terms of preparation time, and produce a sufficient yield [20].

The testing details for the long COVID group were the same as those described elsewhere, with a continuous four-minute, two-tone, eyes-closed protocol presenting 200 common tones (1000 Hz) and 40 rare tones (2777 Hz) in random order, once per second [12-13,17]. This protocol was used to acquire both background EEG and evoked ERP data, as discussed below.

WAVi Scan extracts EEG spectral information using standard Fourier transform methods. All trials included automatic artifact rejection, which excluded sections of EEG data with higher than acceptable amplitudes and excessive band frequency activities in the standard EEG bands (Delta, Theta, Alpha, and Beta) on an individual channel basis. Files were also manually inspected to confirm the proper detection of artifacts. Segments of data that contained synchronized eye blinks were also excluded from the study. Finally, spectra were extracted from the background eyes-closed EEG acquired during the auditory oddball ERP, consistent with the previous PPCS study against which we compare here [13].

Study measures

EEG Spectra

Spectral analysis characterizes the frequency composition of EEG, with the alpha band (8-12 Hz) representing the dominant rhythm. We further examine alpha power topography, which in healthy populations (eyes-closed condition) typically exhibits bilateral symmetry and posterior dominance. In PPCS, however, deviations from this pattern occur, including anterior displacement of alpha power and hemispheric asymmetries. These electrophysiological alterations have also been documented in other conditions, such as emotional dysregulation [13,21-23].

In this work, we investigate whether these PPCS spectral physiologies are also present in long COVID. In particular, we calculate the ratio of frontal to occipital alpha microvolt amplitudes using the sum of the magnitudes at each of the alpha band frequencies (8-12 Hz) averaged across the five frontal locations FP1 and F3 (on the left), FP2 and F4 (on the right), and the central Fz and the two occipital locations O1 and O2.

In keeping with previous works, we calculate frontal left-right asymmetries between F3 and F4 as the absolute value of the deviation from 1.0 (symmetry) [13].

EEG Coherence

EEG coherence quantifies frequency-dependent synchronization between electrode pairs, reflecting functional connectivity. Post-mTBI studies demonstrate elevated coherence, particularly in long-range interhemispheric pathways, where some of these abnormalities persist after symptom resolution or a return to normal performance on neuropsychological evaluations [24-27]. It has been posited that concussed participants may recruit additional brain resources to compensate for their inability to produce the necessary level of amplitude, implying that functional recovery may occur faster than physiological recovery [13,24].

Alpha band coherence was extracted using standard methods described elsewhere [12-13,17]. As with the spectral analysis described above, eyes-closed coherence measures were extracted during the same eyes-closed P300 protocol. There are many methods of presenting coherence, but with $\frac{N \times (N - 1)}{2} = 171$ pairs to choose from, care must be taken not to overfit the data. This study employs an established connectivity quantification method [13,18-19], where strong functional connections are defined as those exceeding two standard deviations above the mean of a reference control dataset. Our control cohort exhibited a mean of three connections above this threshold (out of 171 possible), consistent with expectations for a Gaussian distribution. This approach enables the standardized quantification of network alterations in conditions such as post-viral fatigue (e.g., long COVID) and PPCS, where hyperconnectivity patterns are anticipated. The method accounts for inter-individual variability in both connection strength and spatial distribution of affected pathways.

It should be noted that coherence, like many other EEG metrics, is susceptible to artifacts, and the raw EEG must be visually inspected to ensure that artifacts don't affect long- or short-distance coherences. We used alpha band coherence because it is less susceptible to motion artifacts and/or muscle tension than the other bands, and because alpha often accounts for the most significant amount of power in the EEG band, thus yielding the most robust coherence estimates.

Evoked EEG

The final set of measures involves an auditory oddball ERP, a common protocol that measures the brain's response to common rare tones. The brain typically identifies the rare as different by around 300 ms after the delivery of the oddball stimulus, presenting as a positive EEG voltage, hence the name P300. The amplitude of the P300 response is considered to be proportional to the number of attentional resources devoted to detecting the rare tone, where larger amplitudes and smaller latencies (faster processing speeds) have been associated with superior information processing. The P300 amplitude has been observed to decline after a concussion and with PPCS, whereas the latency remains a more stable trait [12-13,19].

In this work, the depth (P300V) was extracted from the mean amplitude of the response to all stimuli. The amplitudes of the P300 components reported here were measured by identifying the average positive extremum in the latency range of 240-500 ms, relative to the average of the first 16 ms post-stimulus, with baseline correction using the 100-ms pre-stimulus period. Here, we report the average P300 µV from the six C-P scalp sites (C3, CZ, C4, P3, Pz, P4).

Statistical analysis

Following the procedure of the previous PPCS study [13], the mean of the metrics of interest for the long COVID group was compared to that of the control group using unpaired two-sample t-tests, with a p-value cutoff of 0.05 and a minimum meaningful effect size of Cohen's d > 0.50 [28].

## Results

Initial evaluations

At their initial baseline evaluation, these long COVID patients exhibited similar symptom profiles to those often seen in PPCS cases (Table 1), with cognitive fatigue being the most commonly reported symptom.

**Table 1:** CP symptom screening in patients with post-viral fatigue, rated on a scale from 0 (none) to 3 (severe) CP: cognitive performance

Profile category	Mean symptom score
Cognitive fatigue	1.8
Mood	1.5
Sleep	1.4
Visual	0.9
Cervical	0.9
Vestibular	0.8
Migraine	0.8

**Figure 2:**
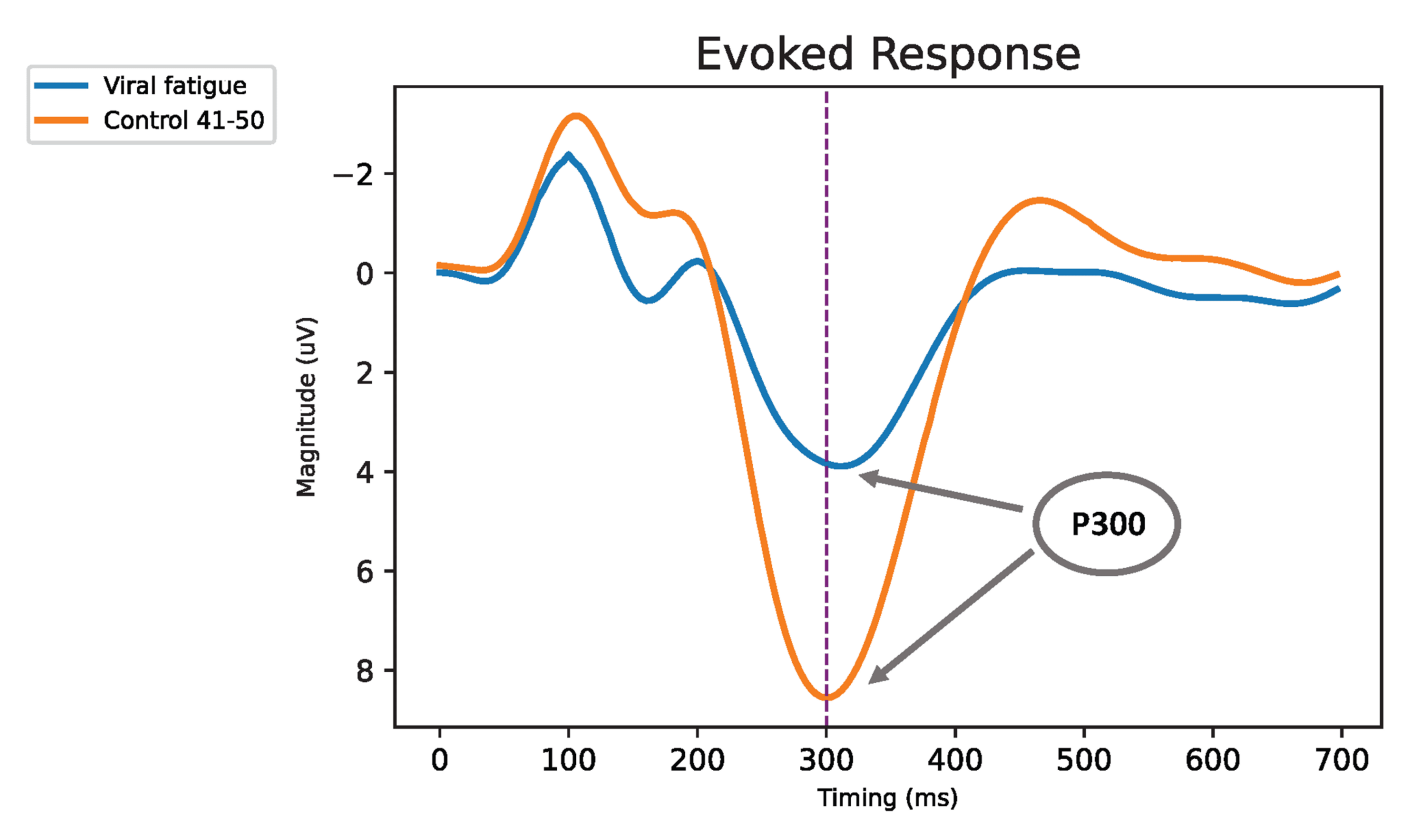
Evoked response amplitude (central-parietal) as a function of time after the delivery of rare stimuli, averaged across control and long COVID (viral fatigue) groups, showing a clear reduction in amplitude but no change in latency of the P300 cognitive response COVID: coronavirus disease

While no reduction in TMT times was reported, physical reaction times were suppressed in both PPCS and long COVID (Table 2). Electrophysical deficits were also seen in both the long COVID and PPCS populations (Table 2), where cognitive resources, as measured by the P300 evoked response amplitude, were diminished (Figure 2) with no reduction in latency. Here, 70% of the long COVID patients were one standard deviation below that observed for the controls in amplitude (16% expected).

**Table 2:** EEG, ERP, and performance metrics in post-viral fatigue, PPCS, and control groups COVID: coronavirus disease 2019, SD: standard deviation, CohD: Cohen's D, EEG: electroencephalography, ERP: event-related potential, PPCS: persistent post-concussion symptom, OP: occipital-parietal Bold: significant p-value difference from control

Metric	Control (N=70)	Long COVID (N=31)	PPCS (N=64)
Performance			
Reaction time (SD)	318 (87) ms	403 (122) ms	387 (118) ms
P-value (CohD)	-	<0.001 (0.8)	<0.001 (0.7)
ERP			
P300 voltage (SD)	9 (4) uV	5 (3) uV	6 (4) uV
P-value (CohD)	-	<0.001 (1.3)	<0.001 (1.0)
P300 latency (SD)	290 (35) ms	286 (32) ms	294 (34) ms
P-value (CohD)	-	0.6 (0.1)	0.6 (0.1)
Spectral			
Frontal/OP a amplitude (SD)	0.8 (0.3)	1.1 (0.4)	1.0 (0.4)
P-value (CohD)	-	0.001 (0.7)	<0.001 (0.7)
Frontal asymmetry	0.05 (0.10)	0.15 (0.11)	0.38 (0.51)
P-value (CohD)	-	<0.001 (0.9)	<0.001 (0.9)
Alpha-band connectivity			
# coherence connections > 2 s	3.4 (1.8)	4.8 (5.0)	8.9 (7.4)
P-value (CohD)	-	0.05 (0.4)	<0.001 (1.0)

The alpha amplitude in the long COVID group, as in PPCS [13], trended toward reduction in occipital regions, accompanied by a frontal increase, as illustrated in Figure 1 (top), and a marked trend of alpha asymmetry as measured by the frontal alpha amplitude ratio. Concerning coherence differences, as in PPCS, the long COVID group showed increased coherence in the alpha band compared to the control.

Follow-up evaluations

**Figure 3:**
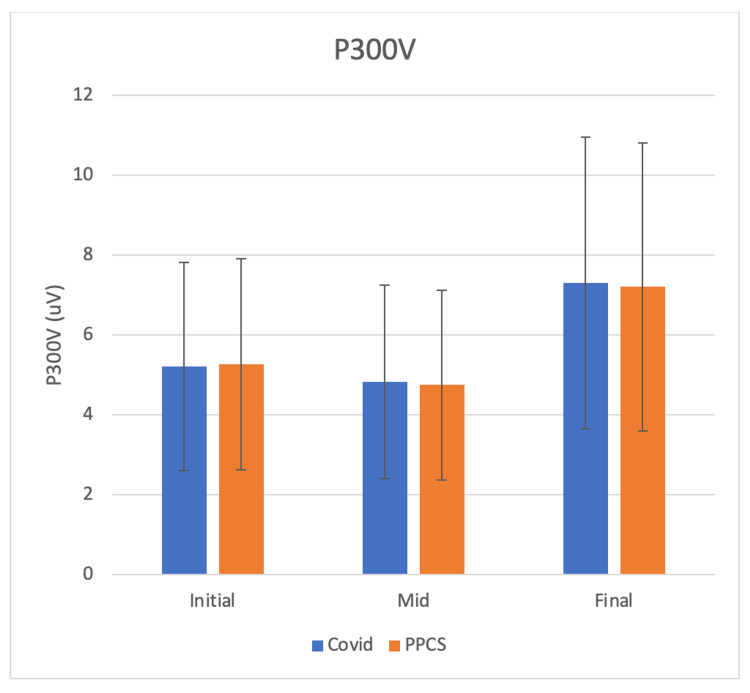
Trajectories of P300 evoked response amplitudes measured from intake through stages of symptom improvement. Both PPCS and long COVID follow a similar path toward P300 normalization PPCS: persistent post-concussion symptoms, COVID: coronavirus disease 2019

Patients with long COVID-related cognitive fatigue received multi-disciplinary management protocols similar to those for PPCS patients within the same clinic. Multi-disciplinary treatments in these cohorts included active rehabilitation protocols, physical therapy, occupational therapy, and cognitive behavioral therapy, administered over 6-28 months (mean duration: 10.5 months). We compared ERP trajectories in 21 patients with long COVID versus 24 patients with PPCS (Figure 3) across serial assessments. Intermediate testing was conducted during the ongoing symptoms, while final testing at 20 ± 8 months post-baseline captured an improved clinical status.

At the intermediate assessment, neither the long COVID nor the PPCS cohorts demonstrated symptomatic improvement, as reflected by stable P300V values. By the final visit, however, both groups exhibited significant symptom reduction, which was paralleled by a marked increase in P300V (p < 0.05, Cohen’s d > 0.5). These improvements were correlated with clinical improvements over time, as measured by the total symptom score using the CP screen tool. Also of note, the number of excess coherence locations changed from six on the initial and mid tests to the more expected three on the final tests.

## Discussion

This study aims to characterize the electrophysiological abnormalities underlying cognitive symptoms in patients with long COVID. By comparing these individuals against both healthy controls and PPCS cohorts, who report similar cognitive complaints, we seek to identify objective electrophysiological signatures that complement clinical symptom profiles.

In the long COVID group, as in the PPCS cohort, we observed a significantly reduced P300 amplitude, indicative of diminished cognitive resources and consistent with patients’ subjective reports. This persistent reduction in P300 amplitude in both groups aligns with the cognitive fatigue and “brain fog” frequently described by affected individuals [11]. Despite the presence of psychosocial confounders and the heterogeneity of the patient population, 70% of long COVID patients exhibited reduced P300 amplitude, compared to only 16% of controls. Notably, this electrophysiological deficit resolved following several months of multi-disciplinary neurorehabilitation in both PPCS and long COVID patients. These findings may reflect underlying neuroinflammation, vascular complications, or metabolic disruptions previously identified through neuroimaging [4,9]. However, it is essential to note that the resolution of symptoms and functional (ERP) measures may not temporally coincide with the normalization of imaging findings.

We observed a frontal alpha power shift in long COVID patients with post-viral fatigue, mirroring patterns previously documented in PPCS cohorts. While the neurophysiological basis of this shift requires further investigation, preliminary evidence suggests it may represent a distinct electrophysiological phenotype [29]. Both long COVID and PPCS groups exhibited significantly greater alpha asymmetry than controls (p < 0.01), a pattern potentially linked to emotional regulation deficits commonly reported in these populations [21-23].

Both long COVID and PPCS cohorts exhibited increased alpha-band coherence, consistent with prior PPCS literature. In PPCS, elevated long-range coherence emerges during subacute recovery phases (absent acutely), suggesting neuroplastic reorganization. While the mechanistic basis of long COVID remains unclear, similar increases may reflect compensatory processes following neural injury, as supported by diffusion imaging evidence of microstructural damage. This interpretation is reinforced by our observation of reduced excess coherence at final assessment (vs. baseline), which paralleled symptomatic improvement in long COVID patients.

Finally, note that while the physical reaction time decreased, performance on the TMT was strong in long COVID, consistent with some studies but not others [4,5]. This highlights the diagnostic challenge clinicians face when patient-reported symptoms lack correlates in standard neuropsychological testing. Crucially, our study demonstrates objective electrophysiological abnormalities that validate brain-related complaints in both long COVID and PPCS cohorts.

Implications

Analysis of the long COVID cohort reveals electrophysiological abnormalities consistent with PPCS patients, correlating with shared cognitive symptom profiles. These findings demonstrate measurable neurophysiological dysfunction in long COVID. Moreover, similar intervention strategies may be employed. With evidence of neurophysiological dysfunction such as frontal alpha, coherence, and P300 amplitude, the results suggest that the combined use of EEG and ERP can help objectively assess long COVID diagnosis, recovery, and prognosis to aid in the subjective assessment of patients with ongoing complaints of persistent symptoms and help support decision-making in care.

Limitations

Potential limitations of the study include the fact that patient data were collected from clinical examinations, which, by nature, creates a heterogeneous population and variable time course in disease (i.e., wide ranges in chronicity and morbidity). While the neurophysiological findings matched symptomology, the small sample size and the patient self-selection may limit the generalizability of this clinic-based sample. Furthermore, the strong correlations between long COVID and PPCS versus the control group support the notion that imaging studies do not directly address causation. Finally, while no gender differences were observed, the sample size was insufficient to make any gender-related comparisons.

## Conclusions

These findings suggest that the electrophysiological abnormalities observed in PPCS are also present in long COVID. Such abnormalities may reflect cognitive fatigue, a symptom commonly reported in both conditions. Notably, the amplitude of the P300 component of the ERP, an index of cognitive processing, increased in both the PPCS and long COVID cohorts as symptoms resolved. These neurophysiological markers may help clinicians identify affected individuals and facilitate earlier, evidence-based interventions, which could be especially valuable in the context of confounding psychosocial factors.
